# The Insertion of Intrauterine Devices in the Immediate Postpartum Period Remains an Important Missed Opportunity to Prevent Unplanned Pregnancies in Brazil

**DOI:** 10.1055/s-0043-1774737

**Published:** 2023-10-16

**Authors:** Adalberto Kiochi Aguemi, Mirna Namie Okamura, Cristina Aparecida Falbo Guazzelli, Maria Regina Torloni

**Affiliations:** 1 Secretaria Municipal de Saúde de São Paulo, São Paulo, SP, Brazil; 2 Escola Paulista de Medicina, Universidade Federal de São Paulo, São Paulo, SP, Brazil

Unplanned pregnancy continues to be a public health problem in Brazil, particularly for the most vulnerable segment of the population. According to data from the Birth in Brazil study, 55% of pregnancies are unplanned, a proportion that reaches 65.3% among adolescents. Although the copper IUD is a highly effective long-term contraceptive method, it is still little used in Brazil for several reasons, including access barriers in outpatient clinics. The offer of IUD insertion immediately after childbirth or abortion is a window of opportunity to avoid unplanned pregnancies.

In 2017, the Ministry of Health launched a project to expand access to this method by encouraging the offer and insertion of copper IUDs in the immediate postpartum and post-abortion periods in Brazilian maternity hospitals for all women willing to use this contraceptive. Despite this initiative, in 2021 the postpartum IUD insertion rate in Brazilian maternity hospitals was less than 1.5% compared with 18% in Mexico in 2014, 1 where the unplanned pregnancy rate was 36%. These data show the need for advances in existing strategies to expand the use of IUDs in Brazilian maternity hospitals, including a continuous public policy by the Ministry and Departments of Health, in addition to studies on the various factors involved in this process.

A study conducted in Brazilian maternity hospitals on the knowledge, attitude and practice of physicians in the use of IUDs in the immediate postpartum and post-abortion periods is published in this issue of RBGO, 2 and points to some of the barriers and facilitators present in our environment. For example, ∼42% of physicians said they had not received any training on IUD insertion in the immediate postpartum (IPP) or immediate post-abortion (IPA). In the previous 12 months, 19.7%, 22.8% and 53.5% of respondents stated they had not inserted an IUD during a cesarean section, immediately after a vaginal delivery and an abortion, respectively. However, this proportion may be lower in Brazilian maternity hospitals, as 95% of maternity hospitals in the study were teaching hospitals. More than 70% of participants consider women's resistance to the method as an important or very important barrier to IUD insertion in the IPP or IPA periods in the public hospital where they work. More than 60% pointed to the unavailability of copper IUDs in the obstetric center and the lack of hospital protocols as important or very important barriers to IUD insertion in the IPP or IPA periods. Other important or very important barriers mentioned by most participants were the lack of experience of physicians and fear of IUD expulsion (in insertions after vaginal or cesarean deliveries), fear of infection or perforation (in insertions after vaginal delivery or abortion) and lack of support from hospital managers (for IUD insertion in the IPA period).

These findings indicate the need to reassess and improve the quality of training currently offered by educational institutions and the involvement of managers to offer refresher courses and training. The addition of a practical training module, including a clinical demonstration (on real patients) and tutor supervision in the obstetrics center for a few days or weeks after the theoretical module could reduce the lack of experience and increase the confidence of physicians in IUD insertion in the IPP and particularly in the IPA period in Brazilian public hospitals. The involvement, performance and support of clinical directors and hospital managers are essential to overcome the main organizational barriers to IUD insertion in the IPP or IPA periods reported by study participants.

It is important that physicians guide and clarify the woman's and partner's doubts during antenatal care about the possibility of IUD insertion in the IPP period, thus increasing the possibility of adherence to this contraceptive method.

Reducing unplanned pregnancies is a priority for entities and health professionals committed to improving maternal and child indicators. The challenge is to change this reality to visualize a promising future.
